# Molecular and Clinical Heterogeneity in Resected Biliary Tract Cancer: A Single-Center Analysis of Treatment Patterns and Oncologic Outcomes

**DOI:** 10.3390/cancers18213472

**Published:** 2026-10-27

**Authors:** Yawen Dong, Sophia Petschnak, Matthias Novotny, Francesca Notte, Madita Tschoegl, Edanur Cenan, Jonas Santol, Mariel Gramberger, Mohamed Salem, Alexander Kupis, Rebecca Thonhauser, Niccolò Surci, Josef Friedl, Thomas Hoblaj, Thomas Gruenberger

**Affiliations:** 1 Department of Surgery, HPB Center, Clinic Favoriten, Health Network Vienna and Sigmund Freud Private University, 1100 Vienna, Austria; yawen.dong@gesundheitsverbund.at (Y.D.); francesca.notte@gesundheitsverbund.at (F.N.); madita-magdalena.tschoegl@gesundheitsverbund.at (M.T.); edanur.cenan@gesundheitsverbund.at (E.C.); jonas.santol@gesundheitsverbund.at (J.S.); mariel.gramberger@gesundheitsverbund.at (M.G.); mohamed.salem@gesundheitsverbund.at (M.S.); alexander.kupis@gesundheitsverbund.at (A.K.); rebecca.thonhauser@gesundheitsverbund.at (R.T.); niccolo.surci@gesundheitsverbund.at (N.S.); josef.friedl@gesundheitsverbund.at (J.F.); thomas.hoblaj@gesundheitsverbund.at (T.H.); 2 Institute of Clinical Pathology, Molecular Pathology and Microbiology, Clinic Favoriten, Health Network Vienna, 1100 Vienna, Austria; sophia.petschnak@gesundheitsverbund.at; 3 Department of Surgery, University Hospital Wiener Neustadt, 2700 Wiener Neustadt, Austria; matthiasjohannes.novotny@wienerneustadt.lknoe.at

**Keywords:** biliary tract cancer, cholangiocarcinoma, gallbladder cancer, molecular profiling, next-generation sequencing, surgical resection, survival

## Abstract

Background/Objectives: Biliary tract cancer (BTC) comprises anatomically and molecularly distinct diseases, yet real-world data integrating molecular testing with clinicopathological characteristics and outcomes after resection remain limited. We evaluated clinical and molecular heterogeneity across the major BTC subtypes in a contemporary surgical cohort. Methods: Consecutive adults undergoing curative-intent resection for intrahepatic (iCCA), perihilar (pCCA), distal (dCCA) cholangiocarcinoma, or gallbladder cancer (GBC) at a single Vienna center between 2019 and 2025 were analyzed at the patient level using the first qualifying resection as the index procedure. Molecular testing (52-gene Oncomine Focus; 50-gene Precision/Genexus), perioperative and pathological outcomes, recurrence, and overall survival (OS) were assessed. Results: The cohort included 145 patients (73 iCCA, 24 pCCA, 22 dCCA, 26 GBC). Molecular profiling was available in 103 patients (71%). IDH1/2 and FGFR2 alterations were confined to iCCA, whereas KRAS alterations were most frequent in pCCA and dCCA. Major morbidity occurred in 54/145 patients (37%) and was highest in pCCA (16/24, 67%); 17 Grade V events were documented. Median OS was 40.0 months and did not differ significantly across anatomical subtypes (log-rank *p* = 0.250). Cumulative incidence of recurrence likewise did not differ significantly across subtypes when death without prior recurrence was treated as a competing event (Gray’s test *p* = 0.163). Conclusions: The principal contribution of this real-world cohort is the integration of routine molecular testing with surgical, pathological, treatment, perioperative, and longitudinal outcome data across resected BTC subtypes. Assay coverage and testing selection must be considered when interpreting molecular findings generated in routine practice.

## Simple Summary

Biliary tract cancers arise in different parts of the biliary system and vary in their clinical behaviors and molecular features. We studied 145 patients who underwent curative-intent surgery for intrahepatic, perihilar, distal, or gallbladder cancer between 2019 and 2025. Molecular profiling was available in 71% of patients and showed distinct patterns across tumor sites, including IDH and FGFR2 alterations in intrahepatic tumors and more frequent KRAS alterations in perihilar and distal tumors. Testing was performed within an evolving routine diagnostic workflow using two targeted NGS panels with different gene coverage. In the full cohort, median overall survival was 40.0 months. When recurrence was analyzed with death without prior recurrence treated as a competing event, recurrence did not differ significantly across anatomical subtypes. Major postoperative morbidity occurred in 37% of patients and was highest in the perihilar subgroup. These findings show the value of integrating molecular information with surgical, pathological, perioperative, and longitudinal clinical data while recognizing the limitations of non-uniform real-world testing.

## 1. Introduction

Biliary tract cancer (BTC) comprises a heterogeneous group of malignancies arising from the intrahepatic and extrahepatic bile ducts and the gallbladder. Intrahepatic cholangiocarcinoma (iCCA), perihilar cholangiocarcinoma (pCCA), distal cholangiocarcinoma (dCCA), and gallbladder cancer (GBC) represent anatomically and clinically distinct entities with differences in presentation, management, and prognosis [1,2,3].

For most patients with localized BTC, surgical resection remains the principal potentially curative treatment [1,2,3]. Nevertheless, recurrence after resection is common, and long-term outcomes vary according to anatomical subtype, pathological stage, lymph node involvement, resection margin status, and other tumor-related factors. The surgical procedures required to achieve complete resection also differ substantially across BTC subtypes, ranging from liver resection for iCCA to major hepatectomy with extrahepatic bile duct resection for pCCA, pancreaticoduodenectomy for dCCA, and extended cholecystectomy or hepatic resection for GBC [1,2,3]. Perioperative outcomes and subsequent oncologic treatment therefore need to be interpreted within the context of the respective anatomical subtype.

Molecular profiling has further demonstrated that BTC encompasses biologically distinct diseases. FGFR2 fusions or rearrangements and IDH1 mutations are predominantly associated with iCCA, whereas KRAS alterations are more frequent in extrahepatic cholangiocarcinoma, and TP53 and ERBB2/HER2 alterations occur with differing frequencies across the anatomical BTC subtypes [4]. The identification of clinically relevant alterations has gained increasing importance with the development of biomarker-directed therapies, establishing molecular profiling as an integral component of treatment selection, particularly in advanced disease [1,5].

However, much of the evidence informing precision oncology in BTC derives from patients with advanced or unresectable disease, clinical trial populations, large genomic databases, or studies focused on individual anatomical subtypes [1,4,5]. Integrated real-world data linking molecular profiles with clinicopathological characteristics, surgical treatment, perioperative outcomes, recurrence, and survival across the spectrum of resected BTC remain comparatively limited.

The interpretation of molecular data in retrospective clinical cohorts presents additional challenges. Real-world molecular profiling is heterogeneous with respect to testing frequency, assay selection, genomic coverage, and reporting. A recent multicenter observational study of patients treated in Germany and Austria identified substantial variability in molecular testing practice, including marked variation in the NGS panels used [6]. Tissue availability and quality, pre-analytical factors, DNA- versus RNA-based approaches, and differences in assay coverage may further influence the success and diagnostic yield of molecular profiling [7,8,9]. Molecular testing may also depend on tumor subtype, calendar period, disease recurrence, clinical indication, and the availability of biomarker-directed therapies, potentially resulting in systematic differences between patients who undergo molecular profiling and those who do not.

This distinction is particularly important when interpreting apparently negative molecular findings. A tumor with technically successful molecular testing and no reportable alteration cannot be considered equivalent to a tumor that was never tested or could not be successfully analyzed [7,8,9]. Studies integrating molecular and clinical data should therefore account for testing uptake, assay coverage and success, specimen source, and the timing of molecular analysis.

A consecutive single-center cohort of patients undergoing resection for BTC provides an opportunity to evaluate molecular findings within the broader clinical pathway, including surgical treatment, perioperative outcomes, systemic therapy, recurrence, and survival. The present study was not designed to identify novel genomic drivers, but to characterize how molecular profiling is implemented and interpreted alongside clinicopathological characteristics, perioperative outcomes, treatment patterns, recurrence, and survival in a consecutive real-world cohort undergoing curative-intent BTC resection at a single center in Vienna between 2019 and 2025.

## 2. Materials and Methods

### 2.1. Study Design and Patient Population

This retrospective single-center cohort study included consecutive adult patients who underwent curative-intent surgical resection for histologically confirmed biliary tract cancer (BTC) at the Department of Surgery, Clinic Favoriten, Vienna, Austria, between 1 January 2019 and 31 December 2025.

Eligible tumors were classified according to their anatomical site of origin as intrahepatic cholangiocarcinoma (iCCA), perihilar cholangiocarcinoma (pCCA), distal cholangiocarcinoma (dCCA), or gallbladder cancer (GBC). Patients undergoing completion resection following an incidental diagnosis of GBC were included.

For patients with more than one qualifying resection during the study period, the first curative-intent BTC resection was defined as the index resection and constituted the baseline observation for patient-level analyses. Subsequent resections were not considered independent index cases, and repeat resections performed for recurrent disease were recorded as post-recurrence treatment events.

Patients were excluded if no curative-intent BTC resection was performed or if the final histological diagnosis was not BTC.

The study was conducted in accordance with the Declaration of Helsinki and institutional guidelines for clinical research at Clinic Favoriten, Health Network Vienna. Under the applicable institutional and regulatory framework for this retrospective, non-interventional analysis, formal Ethics Committee review and approval were not required. The requirement for individual informed consent was waived.

### 2.2. Data Collection

Clinical data were obtained from electronic medical records, operative reports, pathology reports, multidisciplinary tumor board documentation, oncology records, radiology reports, and molecular pathology reports. Data were entered into a standardized study database and reviewed for completeness and internal consistency.

Baseline variables included age at index resection, sex, body mass index, American Society of Anesthesiologists physical status, relevant comorbidities, preoperative bilirubin, albumin, CA 19-9, and anatomical tumor subtype. Treatment variables included receipt of neoadjuvant and adjuvant chemotherapy. Pathological, postoperative, molecular, recurrence, and survival data were recorded in relation to the index resection. Preoperative laboratory values were taken from the routine laboratory assessment closest to the index operation, generally from the day before surgery.

### 2.3. Pathological Assessment

Resection specimens from the index operation were assessed according to routine institutional standards, with histopathological terminology based on the World Health Organization Classification of Digestive System Tumours [10]. Pathological variables included in the principal analyses were pathological T category, lymph node status, histological grade, and resection margin status.

To ensure uniform staging across the study period, tumors were staged or retrospectively harmonized according to the eighth edition of the Union for International Cancer Control (UICC) TNM Classification of Malignant Tumours [11]. For comparative analyses, T category was grouped as T1–2 versus T3–4 and histological grade as G1–2 versus G3–4.

An R0 resection was defined as the absence of microscopic tumor at all assessed resection margins, an R1 resection as microscopic tumor involvement of at least one resection margin, and an R2 resection as macroscopically residual disease. Margin categories are reported separately. Liver-side microscopic margins require particular caution in ultrasonic aspirator-assisted hepatic transection (HEPACCS; Söring GmbH, Quickborn, Germany) because the fragmented transection surface may complicate interpretation of an R1 designation.

### 2.4. Molecular Pathology and Biomarker Assessment

Molecular testing was performed as part of routine clinical care at the Department of Pathology, Clinic Favoriten, using formalin-fixed, paraffin-embedded tumor tissue.

Targeted DNA- and RNA-based next-generation sequencing (NGS) was performed as part of routine clinical diagnostics. During the earlier study period, testing was performed using the Oncomine Focus Assay (OFA). The institutional workflow transitioned in late 2024 to the Oncomine Precision Assay on the Genexus System (Thermo Fisher Scientific, Waltham, MA, USA), which became the predominant platform in 2025. The assays differed in gene content and alteration-type coverage; complete panel content is provided in Supplementary Table S5. Published analytical validation and implementation data for the relevant targeted NGS workflows are available [12,13]. Molecular testing was performed within routine quality-controlled laboratory workflows, including regular external genomic quality-assessment/proficiency testing.

For primary-tumor molecular analyses, only molecular results reliably attributable to a patient in the index cohort were included. Molecular analyses performed on recurrent or metastatic tumor specimens were recorded separately and were not attributed to the index primary tumor.

Alterations evaluated in the principal analyses included IDH1, IDH2, FGFR2, KRAS, NRAS, BRAF, ERBB2, PIK3CA, and TP53 where covered by the respective assay. Pathogenic and likely pathogenic alterations and clinically relevant gene fusions or rearrangements were included, whereas variants of uncertain significance were not classified as positive findings [14]. TP53 was not interrogated by OFA and was therefore not analyzed using a full-cohort negative prevalence denominator. Several other BTC-relevant genes, including ARID1A, BAP1, and PBRM1, were not included in either routine assay used during the study period.

Complementary FGFR2 fluorescence in situ hybridization (FISH) and microsatellite instability (MSI) testing were included where available. FGFR2 FISH was used as an orthogonal assay because targeted NGS may have limited sensitivity for some fusion/rearrangement events in routine material. FGFR2 break-apart FISH detects rearrangement-associated signals but cannot determine the fusion partner or establish an expressed functional fusion transcript; targeted RNA-based NGS can identify a fusion partner when detected. The older Oncomine Focus Assay targets predefined fusion configurations, whereas Precision/Genexus incorporates additional detection approaches; assay design and RNA quality may contribute to discordance. Routine histopathologic assessment of tumor-cell content was used for specimen adequacy; a harmonized quantitative genomic tumor-purity variable was not available retrospectively across the full study period.

Alteration prevalence was calculated using the relevant assay-specific evaluable denominator only when the gene or biomarker was covered by the assay used. NGS-detected alterations were reported among appropriately evaluable patients, whereas FGFR2 FISH and MSI results were calculated using their respective evaluable populations. Because NGS panel content varied during the study period, uninformative historical negatives were not treated as wild type for genes absent from the corresponding assay. Molecular profiling uptake was additionally evaluated according to anatomical subtype and study year.

### 2.5. Perioperative Outcomes

Postoperative complications documented during the index hospitalization or during available follow-up within 90 days after the index resection were recorded and graded according to the Clavien–Dindo classification [15]. Major morbidity was defined as Clavien–Dindo grade IIIa or higher. Index-hospitalization complication status was ascertainable for all patients. Vital status at postoperative day 90 could be confirmed in 141 of 145 patients; four patients were discharged alive and subsequently returned to their countries of residence abroad without further reliable follow-up before day 90. The structured study database recorded the highest Clavien–Dindo grade but did not contain a standardized adjudicated cause-of-death field for all postoperative deaths.

### 2.6. Systemic Treatment and Follow-Up

Neoadjuvant chemotherapy was defined as systemic chemotherapy administered before the index resection as part of the curative-intent treatment pathway. Adjuvant chemotherapy was defined as systemic chemotherapy administered following the index resection in the absence of documented recurrent disease. Systemic treatment administered following recurrence was not classified as adjuvant treatment.

Postoperative surveillance was performed according to institutional practice and multidisciplinary tumor board recommendations. Follow-up information was obtained from clinical visits, imaging reports, oncology documentation, and available mortality records. For patients without subsequent institutional follow-up, survival time was censored at the last date on which vital status was documented.

### 2.7. Study Endpoints

The principal study objectives were to characterize molecular profiling uptake and the distribution of molecular alterations across the major anatomical BTC subtypes. Additional analyses evaluated differences in baseline clinicopathological characteristics, use of neoadjuvant and adjuvant chemotherapy, postoperative morbidity and mortality, and oncological outcomes across anatomical subtypes.

Overall survival (OS) was calculated from the date of the index resection to death from any cause. Patients alive at last follow-up were censored on that date. Recurrence was defined as the first documented postoperative recurrence after the index resection. For recurrence-specific analyses, death without prior documented recurrence was treated as a competing event, and patients alive without recurrence were censored at the date of their most recent disease assessment. Recurrence-specific analyses included patients with evaluable postoperative recurrence timing. Conventional recurrence-free survival (RFS), defined as time from the index resection to first documented recurrence or death from any cause, whichever occurred first, was retained as a supplementary analysis.

### 2.8. Statistical Analysis

Continuous variables were summarized as median with interquartile range (IQR), and categorical variables as absolute numbers and percentages using the number of patients with available data as the denominator. Comparisons among iCCA, pCCA, dCCA, and GBC were performed using the Kruskal–Wallis test for continuous variables and the chi-squared test or Fisher’s exact test for categorical variables, as appropriate.

Molecular profiling rates were evaluated according to anatomical subtype and study year. Alteration prevalences were calculated among patients with evaluable results for the corresponding molecular assay and gene coverage. Because genomic coverage differed between NGS platforms during the study period, results were not interpreted as uniform population-level prevalence estimates.

OS was estimated using the Kaplan–Meier method and compared across anatomical subtypes using the log-rank test. Recurrence was analyzed within a competing-risk framework, with first documented recurrence as the event of interest and death without prior recurrence as the competing event; cumulative incidence functions were compared using Gray’s test. The OS analysis included the entire 145-patient index cohort from the date of surgery. Conventional full-cohort RFS and the complementary analysis restricted to patients with confirmed survival to postoperative day 90 were retained in the Supplementary Materials. Survival and recurrence analyses were unadjusted and considered exploratory.

Missing data were not imputed, and variable-specific analyses used the number of patients with available information as the denominator. Details of pathological data availability are provided in Supplementary Table S1. Subtype comparisons were considered exploratory, particularly for the smaller pCCA, dCCA, and GBC groups. All statistical tests were two-sided, and a *p*-value < 0.05 was considered statistically significant. Statistical analyses were performed using IBM SPSS Statistics, version 28.0 (IBM Corp., Armonk, NY, USA) and R version 4.5.3 (R Foundation for Statistical Computing, Vienna, Austria).

## 3. Results

### 3.1. Study Cohort and Baseline Characteristics

**Figure 1:**
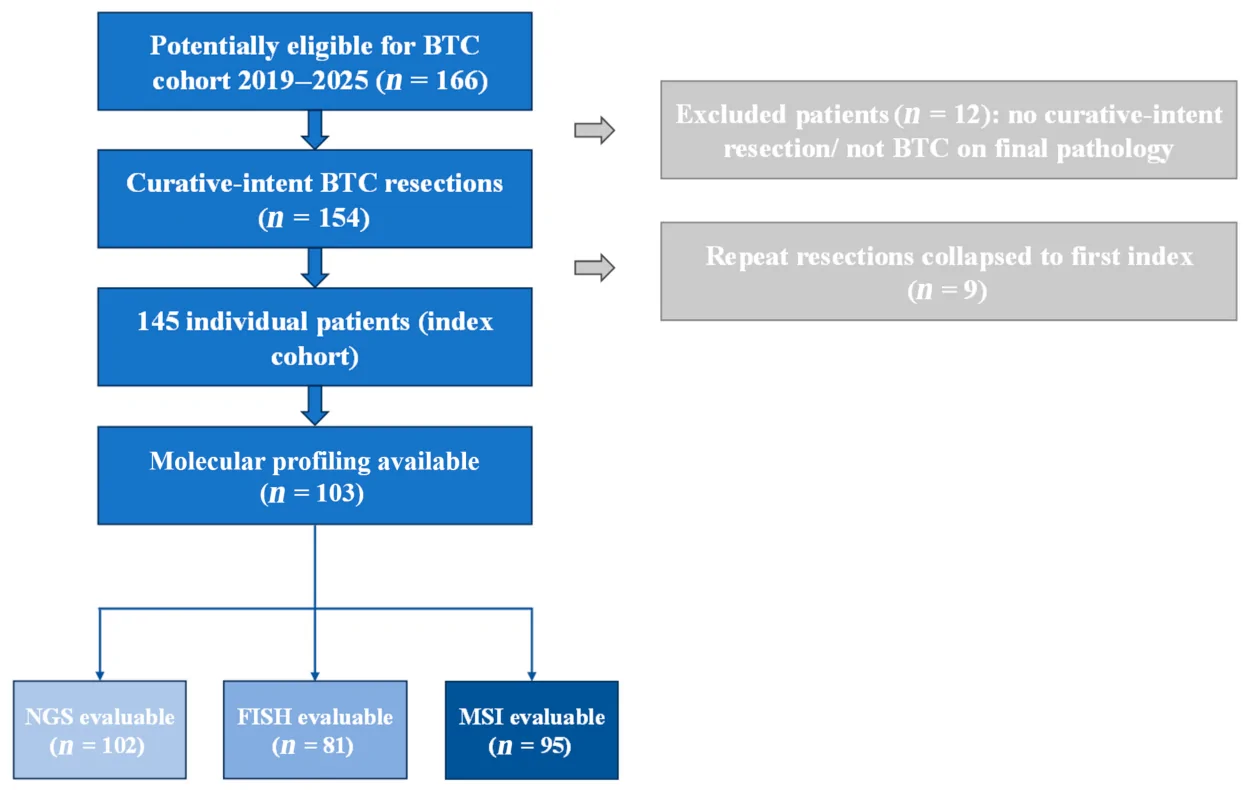
Cohort selection and derivation of the 145-patient index cohort. Blue boxes indicate cohort and molecular testing stages; grey boxes indicate exclusions and repeat resections. Arrows indicate the flow between stages. The NGS, FGFR2 FISH and MSI evaluable subgroups may overlap.

Across seven consecutive years from 2019 through 2025, 166 surgical procedures were identified as potentially eligible for the BTC cohort. Twelve were excluded because no curative-intent BTC resection was performed or the final histological diagnosis was not BTC, leaving 154 curative-intent BTC resections. To establish a patient-level cohort, nine repeat resections were collapsed onto each patient’s first index resection, resulting in 145 unique patients. Baseline, pathological, perioperative, and primary-tumor molecular variables were assigned to the index resection. The cohort comprised 73 patients with iCCA (50.3%), 24 with pCCA (16.6%), 22 with dCCA (15.2%), and 26 with GBC (17.9%) (Figure 1; Table 1).

Median age at index resection was 70.8 years (IQR, 60.9–76.8) and did not differ significantly across anatomical subtypes (*p* = 0.100). Overall, 74 of 145 patients (51%) were male, with the highest proportion among patients with pCCA (79%; *p* = 0.016). Body mass index differed statistically across subtypes (*p* = 0.033), but the absolute differences were modest (median range, 24.8–27.5 kg/m^2^) and were considered descriptive rather than clinically meaningful in isolation. ASA physical status and the prevalence of recorded comorbidities did not differ significantly between groups (Table 1).

Preoperative biochemical profiles differed according to anatomical subtype. Median bilirubin was highest in pCCA at 2.8 mg/dL (IQR, 0.7–4.6), followed by dCCA at 0.9 mg/dL (IQR, 0.5–2.1), compared with 0.5 mg/dL in both iCCA and GBC (*p* < 0.001). Serum albumin was lower in pCCA and dCCA (median 37.0 g/L in both) than in iCCA and GBC (*p* < 0.001), whereas CA 19-9 did not differ significantly (*p* = 0.092). These biochemical differences are interpreted descriptively and may reflect differences in cholestatic burden, inflammation, nutritional status, and preoperative treatment pathways. Neoadjuvant chemotherapy was administered in 21 of 139 evaluable patients (15%), and adjuvant chemotherapy in 80 of 126 (63%), without statistically significant differences across anatomical subtypes (*p* = 0.151 and *p* = 0.162, respectively) (Table 1).

### 3.2. Perioperative and Pathological Outcomes

Postoperative complication status during the index hospitalization was available for all 145 patients. Major morbidity, defined as Clavien–Dindo grade IIIa or higher, occurred in 54 of 145 patients (37%). Major morbidity was observed in 23 of 73 patients with iCCA (32%), 16 of 24 with pCCA (67%), 8 of 22 with dCCA (36%), and 7 of 26 with GBC (27%). Seventeen Grade V events were documented within 90 days, including 5 iCCA, 8 pCCA, 3 dCCA, and 1 GBC patients (Table 2). Cause-specific postoperative mortality was not uniformly captured as a standardized study variable and was therefore not retrospectively adjudicated into potentially inconsistent categories.

Pathological characteristics varied across the anatomical subtypes (Table 3). Among evaluable patients, T3–4 disease was present in 34 of 121 (28%) and node-positive disease in 54 of 117 (46%). Margin status comprised 53 R0, 51 R1, and 4 R2 resections among 108 evaluable patients. Poorly differentiated tumors (G3–4) accounted for 31 of 84 evaluable tumors (37%). Variable-specific denominators reflect available pathological data; further details are provided in Supplementary Table S1.

### 3.3. Molecular Testing

**Figure 2:**
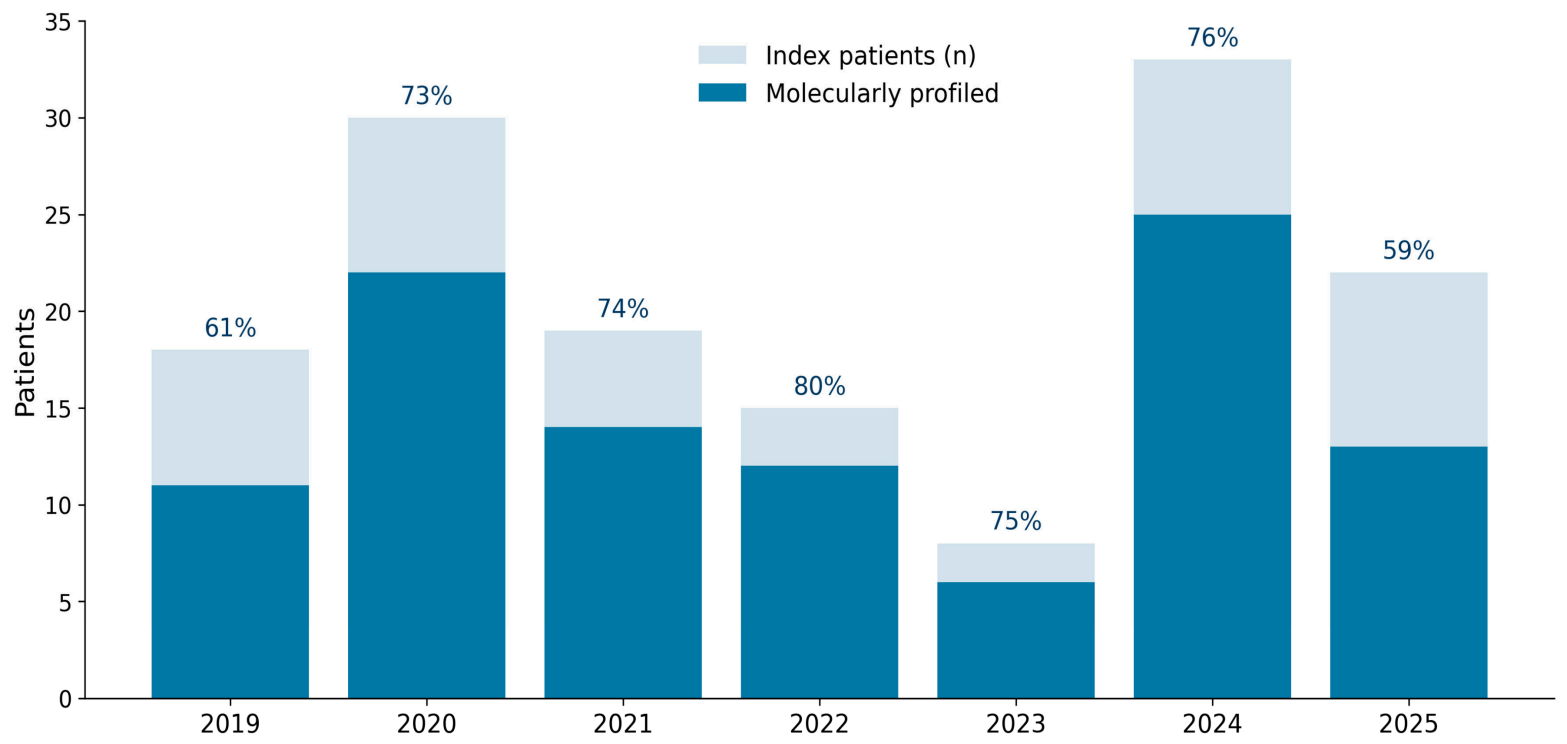
Molecular testing uptake by year.

Molecular profiling was available in 103 of 145 patients (71%). Evaluable results were available for 102 patients by NGS, 81 by FGFR2 FISH, and 95 by MSI testing (Figure 1; Table 4). Profiling uptake varied across anatomical subtypes: 61 of 73 patients with iCCA (84%), 15 of 24 with pCCA (62%), 14 of 22 with dCCA (64%), and 13 of 26 with GBC (50%) were profiled. At least one NGS-detected reportable alteration was identified in 53 of 102 NGS-evaluable tumors (52%) (Table 4). Profiling uptake differed by anatomical subtype (chi-squared *p* = 0.006). Testing also varied descriptively by calendar year (Figure 2; Supplementary Table S4), although the year-by-profiling association was not statistically significant (chi-squared *p* = 0.740). These patterns emphasize that the profiled subgroup should not be treated as a random sample of the surgical cohort.

Annual testing uptake ranged from 59% to 80%, with 11/18 patients profiled in 2019 (61%), 22/30 in 2020 (73%), 14/19 in 2021 (74%), 12/15 in 2022 (80%), 6/8 in 2023 (75%), 25/33 in 2024 (76%), and 13/22 in 2025 (59%) (Figure 2; Supplementary Table S4). This non-monotonic pattern, together with the significant subtype-dependent uptake, underscores selection inherent to routine molecular testing.

### 3.4. Molecular Alteration Landscape

**Figure 3:**
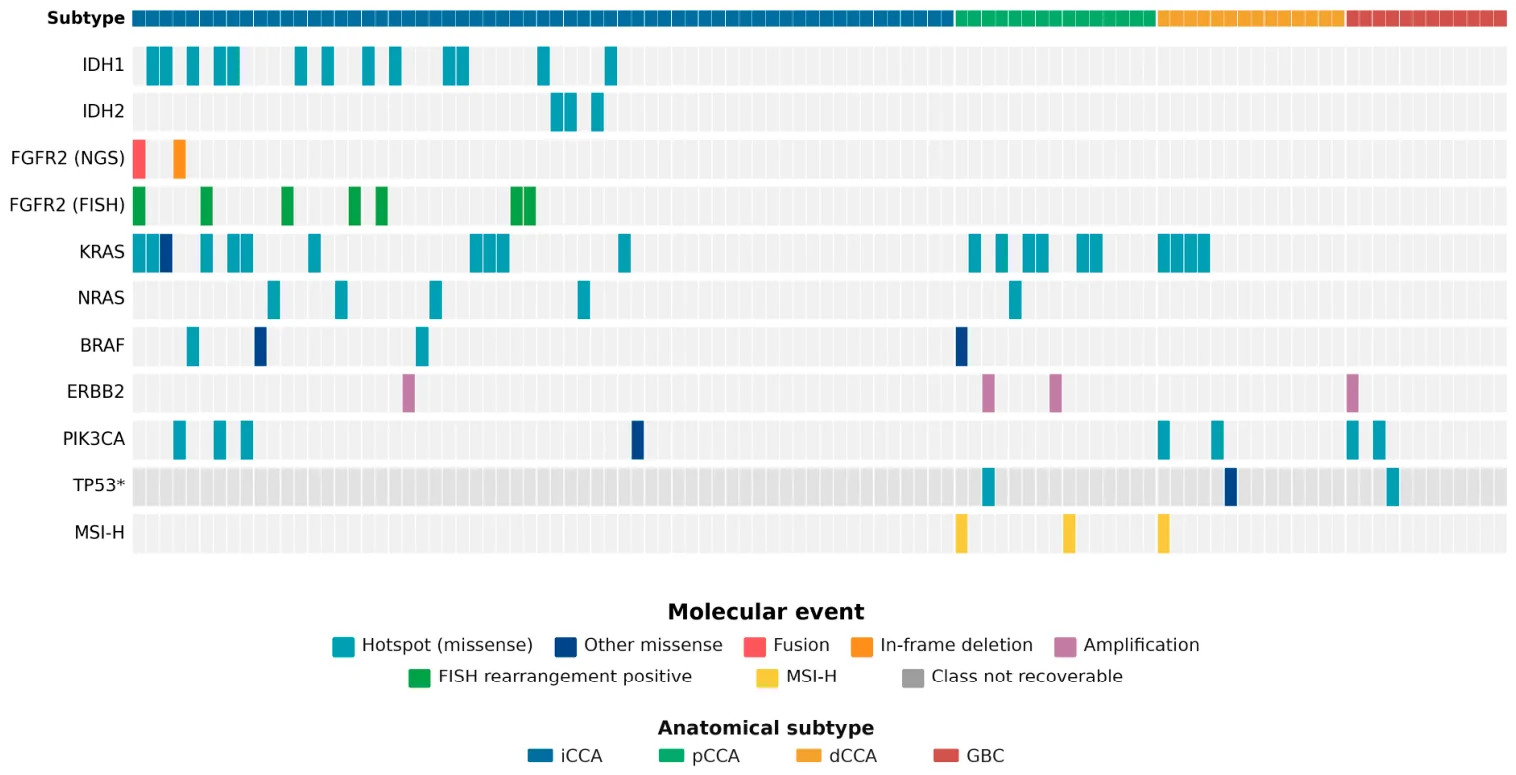
Molecular alteration landscape of NGS-evaluable tumors (*n* = 102). Each column represents one NGS-evaluable index-primary tumor, and columns are grouped by anatomical subtype. Molecular events are coded by alteration class; FGFR2 FISH and MSI-H are shown as orthogonal biomarker results. Assay context: the Oncomine Focus Assay (OFA; 52 genes; DNA/RNA hotspot, copy-number and fusion targets) was used during the earlier study period, whereas the Oncomine Precision/Genexus assay (50 genes, including TP53) was introduced in late 2024 and became predominant in 2025. TP53 was not interrogated by OFA, so absence of a TP53 call in OFA-tested samples must not be interpreted as TP53 wild type. ARID1A, BAP1, and PBRM1 were not included in either assay. Complete gene and alteration-type coverage is provided in Supplementary Table S5. Exact event class is shown where recoverable; otherwise, the call is marked as class not recoverable.

**Figure 4:**
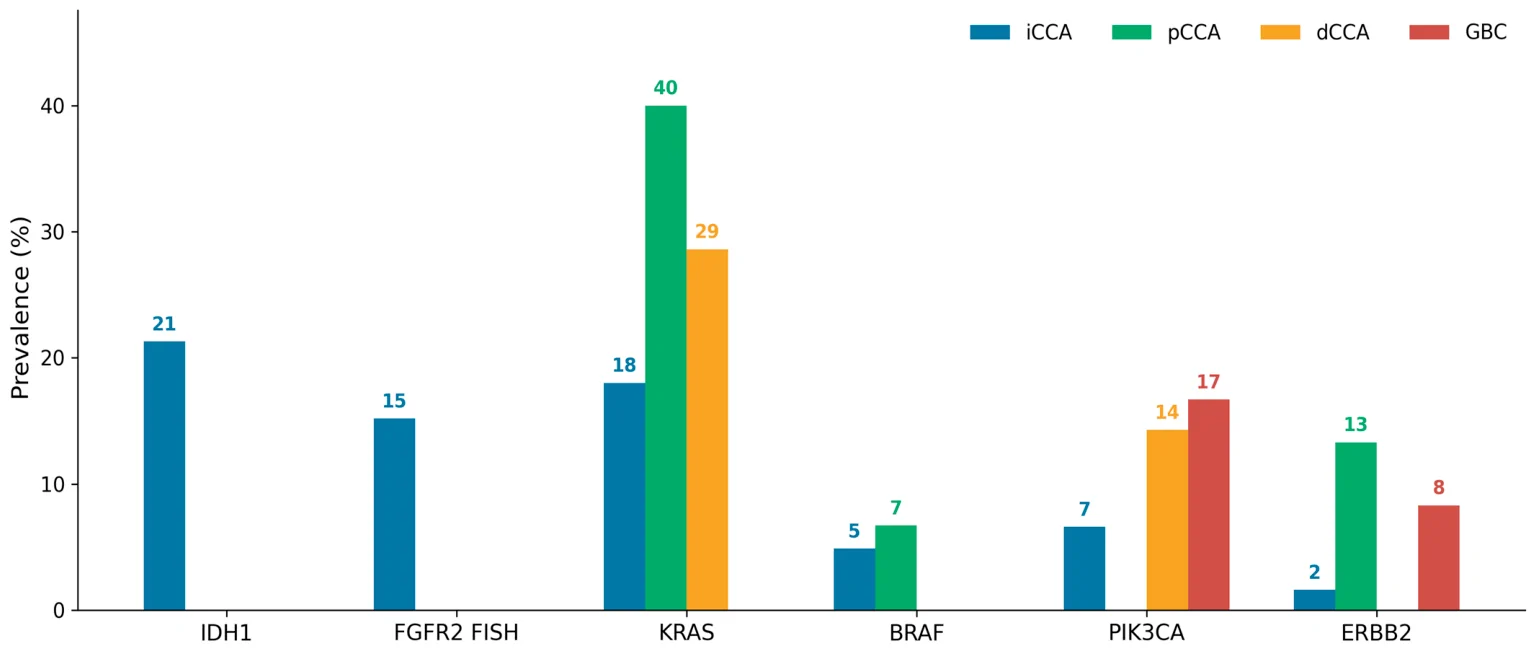
Selected alteration prevalence by subtype.

The molecular landscape differed according to anatomical subtype (Figure 3 and Figure 4; Table 5). Among NGS-evaluable tumors, KRAS was the most frequently identified alteration, occurring in 21 of 102 tumors (20.6%), followed by IDH1 in 13 of 102 (12.7%) and PIK3CA in 8 of 102 (7.8%). Figure 3 distinguishes molecular event classes and makes assay-coverage limitations explicit. Complete OFA versus Precision/Genexus gene coverage is provided in Supplementary Table S5. The two targeted panels (Oncomine Focus, 52 genes; Oncomine Precision/Genexus, 50 genes) did not interrogate ARID1A, BAP1 or PBRM1; TP53 was covered only by Precision/Genexus. Patient-level reportable molecular alterations, corresponding NGS platforms and molecular pathology accession years for the 53 alteration-positive NGS-evaluable tumors are provided in Supplementary Table S9.

IDH1 alterations were confined to iCCA and were identified in 13 of 61 NGS-evaluable iCCA tumors (21.3%). IDH2 alterations were likewise restricted to iCCA and occurred in 3 of 61 tumors (4.9%). FGFR2 rearrangements detected by FISH were identified in 7 of 46 evaluable iCCA tumors (15.2%) and were not detected in pCCA, dCCA, or GBC. Targeted review of the seven FISH-positive iCCA tumors showed that six were negative for an FGFR2 event by NGS and one was concordant, with an FGFR2(17)-CASP7(2) fusion detected by NGS (Supplementary Table S6). This pattern supports the complementary local use of FISH in cases in which targeted NGS does not identify an FGFR2 rearrangement.

KRAS alterations were identified in 11 of 61 iCCA tumors (18.0%), 6 of 15 pCCA tumors (40.0%), and 4 of 14 dCCA tumors (28.6%); no KRAS alterations were identified among the 12 NGS-evaluable GBC tumors. Four iCCA tumors showed uncommon KRAS co-alterations with IDH1 or FGFR2, including one KRAS/FGFR2 case with positive FGFR2 FISH. The corresponding alterations are detailed in Supplementary Table S7. ERBB2 alterations were detected in 4 of 102 NGS-evaluable tumors (3.9%), PIK3CA alterations in 8 of 102 (7.8%), and BRAF alterations in 4 of 102 (3.9%) (Table 5). TP53 was not uniformly interrogated because it was absent from OFA; TP53 calls are therefore shown descriptively without a full-cohort prevalence denominator.

MSI-high status was identified in 3 of 95 evaluable tumors (3.2%), comprising 2 of 14 pCCA tumors (14.3%) and 1 of 13 dCCA tumors (7.7%). No MSI-high tumors were identified among evaluable iCCA or GBC tumors.

### 3.5. Overall Survival and Recurrence

**Figure 5:**
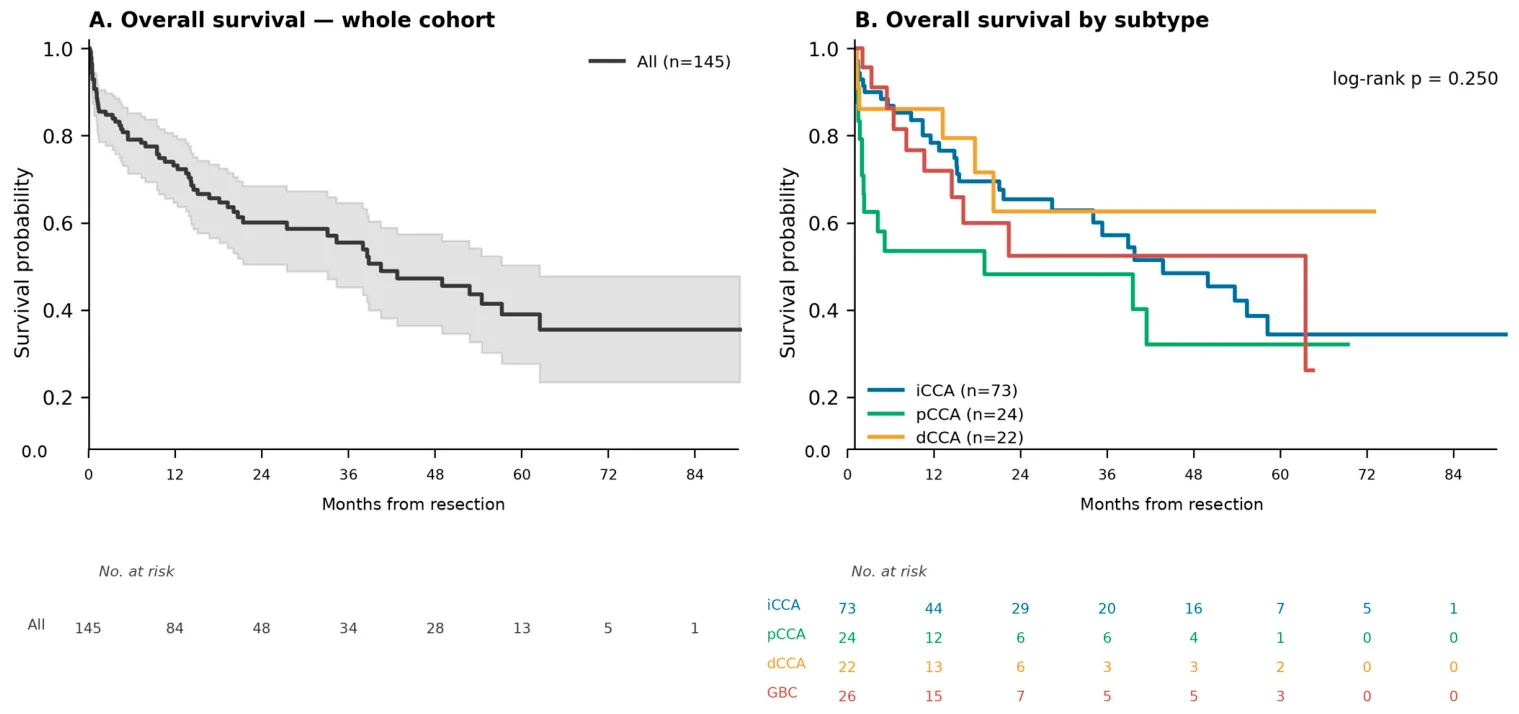
Overall survival from the date of index resection in the full 145-patient cohort: (**A**) whole cohort and (**B**) by anatomical subtype. In panel A, the grey shaded band indicates the 95% confidence interval around the Kaplan–Meier survival estimate; in panel B, the red curve indicates gallbladder cancer (GBC). Numbers at risk are shown below the plots.

In the full 145-patient cohort, median OS from the index resection was 40.0 months (Figure 5; Table 6). Median OS was 42.2 months for iCCA, 17.8 months for pCCA, 61.8 months for GBC, and was not reached for dCCA. OS did not differ significantly across anatomical subtypes on unadjusted Kaplan–Meier analysis (log-rank *p* = 0.250).

**Figure 6:**
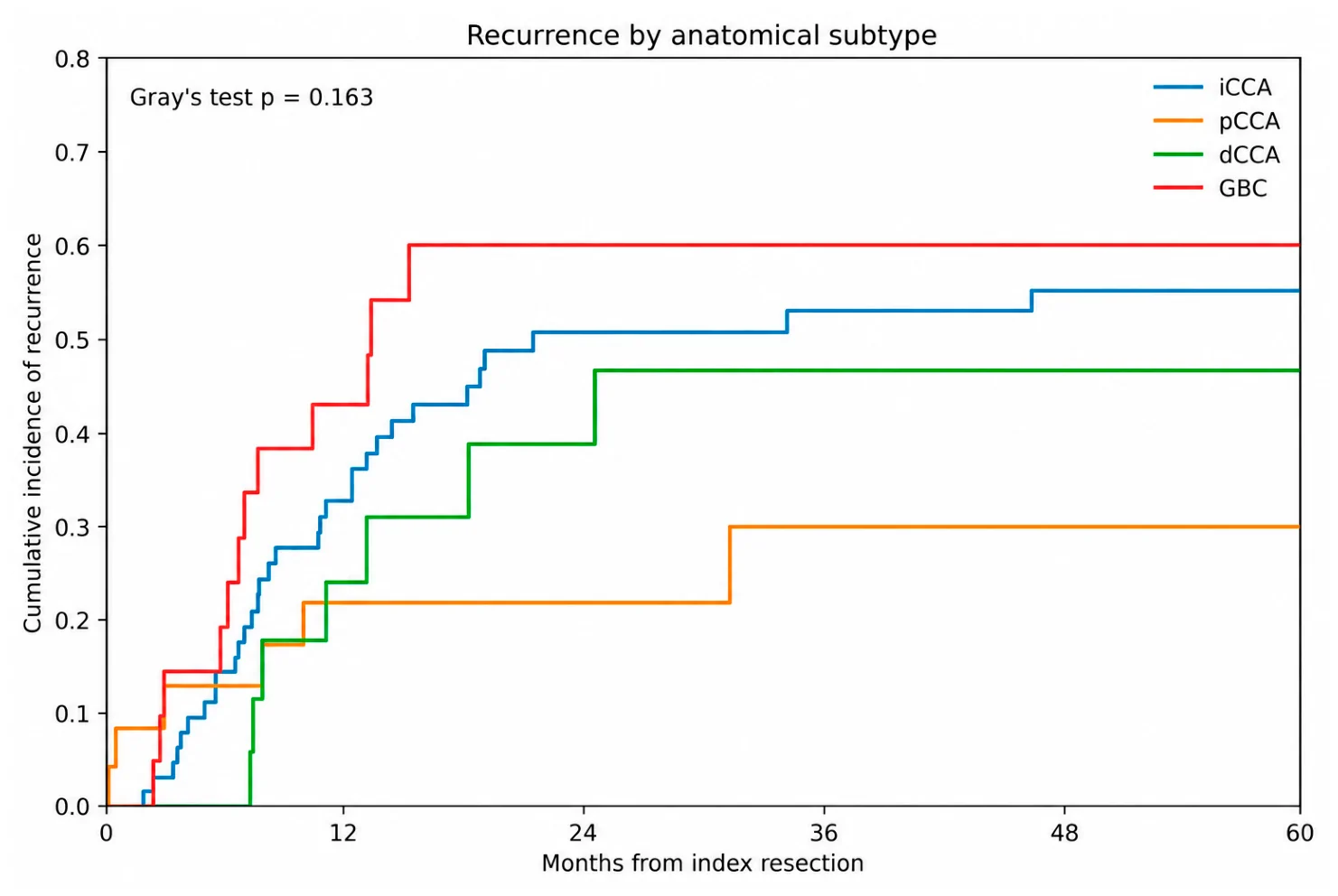
Cumulative incidence of first postoperative recurrence by anatomical subtype. Death without prior documented recurrence was treated as a competing event; curves were compared using Gray’s test.

The cumulative incidence of recurrence did not differ significantly across anatomical subtypes (Gray’s test *p* = 0.163) (Figure 6). At 12, 24, and 36 months, cumulative recurrence incidences were 32.7%, 50.7%, and 52.9% for iCCA; 21.7%, 21.7%, and 29.8% for pCCA; 23.9%, 38.7%, and 46.6% for dCCA; and 43.0%, 60.0%, and 60.0% for GBC, respectively. The complementary analysis restricted to patients confirmed alive at postoperative day 90 remains available in Supplementary Table S2 and Supplementary Figures S1 and S2. Conventional full-cohort RFS is provided as a supplementary analysis (Supplementary Figure S3; Supplementary Table S8). An exploratory R0-versus-R1 sensitivity analysis among patients with evaluable margin status is provided in Supplementary Figure S4.

## 4. Discussion

In this consecutive cohort of 145 patients undergoing curative-intent resection for BTC, the principal contribution is the integration of routine molecular testing with surgical, pathological, treatment, perioperative, and longitudinal outcome data across all four major anatomical subtypes. The observed molecular patterns were broadly concordant with established BTC biology rather than representing novel genomic associations [16,17,18,19,20]. The study therefore provides a real-world view of how molecular testing is implemented in resected BTC and how assay limitations coexist with clinically important differences in surgical pathways and outcomes.

The molecular distribution remained anatomically patterned, with IDH1/2 and FGFR2 alterations confined to iCCA and KRAS alterations more frequent in extrahepatic cholangiocarcinoma. Interpretation, however, depends on the assay actually used. OFA and Precision/Genexus differ in gene content, TP53 was not included in OFA, and several other BTC-relevant genes, including ARID1A, BAP1, and PBRM1, were not interrogated by either routine assay. Accordingly, apparently negative findings cannot be interpreted uniformly across the study period, and the reported frequencies should be viewed as assay-dependent detection frequencies under evolving routine clinical workflows rather than population-level prevalence estimates.

The clinical relevance of molecular profiling is increasingly evident in advanced BTC, where biomarker-directed therapies are established for selected molecular subgroups [21,22,23,24]. The present study does not demonstrate gene-specific prognostic or predictive effects after resection. Instead, it illustrates practical issues relevant to real-world testing. In particular, six of seven FGFR2 FISH-positive iCCA tumors were negative for a reportable FGFR2 event by targeted NGS. All six discordant tumors were assessed by the Oncomine Focus Assay. Limited fusion coverage or RNA-related technical factors may explain a negative NGS result; conversely, FISH may yield atypical or false-positive interpretations and cannot establish an expressed functional fusion transcript [25,26]. The direction of discordance cannot be resolved without further orthogonal testing (Supplementary Figure S5). The uncommon KRAS/IDH1 and KRAS/FGFR2 co-alterations are interpreted descriptively without implying a distinct biological pattern. Although KRAS and IDH1/FGFR2 alterations generally tend towards mutual exclusivity, occasional co-occurrence has been documented in iCCA [27]. KRAS p.Val14Ile in K01 is classified by OncoKB as likely oncogenic [28]; bulk-sequencing co-detection does not establish shared clonal origin.

When outcomes were analyzed from the date of surgery in the full cohort, median OS was 40.0 months. Cumulative incidence of recurrence did not differ significantly across anatomical subtypes when death without prior recurrence was treated as a competing event (Gray’s test *p* = 0.163). This recurrence-specific framework distinguishes documented oncologic recurrence from death occurring before recurrence. Conventional RFS, which counts either recurrence or death as an event, is retained as a supplementary analysis together with the complementary 90-day survivor analysis. Given the exploratory nature of the study, no independent prognostic effects are inferred from these unadjusted analyses.

Perioperative risk differed markedly across the anatomical subtypes. Major morbidity occurred in 67% of patients with pCCA compared with 32% with iCCA, 36% with dCCA, and 27% with GBC, and Grade V events were most frequent in pCCA. These crude differences reflect substantially different operative pathways and should not be interpreted as direct measures of tumor biology. The cohort derives from a dedicated HPB surgical practice and includes liver resection, major hepatobiliary resection for pCCA, pancreaticoduodenectomy for dCCA, and extended gallbladder/liver procedures where indicated. Because a standardized cause-of-death variable was not captured for all postoperative deaths, we did not retrospectively assign potentially inconsistent cause categories. The small pCCA, dCCA, and GBC groups also mean that nonsignificant subtype comparisons should not be interpreted as evidence of equivalence.

Conventional RFS, retained in the Supplementary Materials, was shorter than that reported in BILCAP, in which median RFS was 17.5 months with observation and 24.4 months with adjuvant capecitabine [29]. Direct comparisons are limited by differences in trial eligibility, treatment, and case mix. Additional randomized, meta-analytic, real-world, and multicenter data have further characterized postoperative systemic treatment and outcomes after BTC resection [30,31,32,33]. In our cohort, 46% of patients with evaluable nodal status had node-positive disease, consistent with a substantial burden of high-risk pathology. Because conventional RFS includes death as an event, it complements rather than substitutes for the recurrence-specific competing-risk analysis.

Several limitations should be considered. The retrospective single-center design may limit generalizability, as surgical selection, perioperative management, molecular-testing access, pathology workflows, treatment decisions, and follow-up reflect institutional practice. The seven-year study period encompassed changes in molecular diagnostics and oncological standards, and profiling was not randomly distributed by subtype or calendar year. The two NGS assays differed in gene content; TP53 was not interrogated by OFA, while ARID1A, BAP1, and PBRM1 were not included in either assay. Differences between primary and advanced disease and the breadth of genomic profiling provide additional context for interpreting retrospective molecular findings [34,35]. Standardized quantitative tumor purity was not available retrospectively, and cause-specific postoperative mortality was not captured as a uniform adjudicated field. Some pathological variables were incompletely documented, which should be considered when interpreting subgroup comparisons. Finally, the smaller pCCA, dCCA, and GBC cohorts and gene-specific subgroups limit statistical power, and the study was not designed to establish gene-specific prognostic effects or causal treatment associations.

## 5. Conclusions

Resected BTC demonstrates substantial heterogeneity across anatomical site, operative pathway, molecular phenotype, and the implementation of molecular testing. The molecular findings observed in routine clinical practice were broadly concordant with established BTC biology, but their interpretation requires explicit attention to assay coverage and testing selection. The principal contribution of this study is the integration of molecular findings with surgical, pathological, treatment, perioperative, and longitudinal outcome data in a consecutive real-world cohort. More standardized molecular testing and larger multicenter cohorts will be required to define how these dimensions should be integrated in patients with resected BTC.

## Figures and Tables

**Table 1:** Baseline characteristics by subtype (index resection). Values are median (IQR) or *n*/*N* (%).

Characteristic	iCCA	pCCA	dCCA	GBC	Overall	*p*
Age at surgery, y	69.9 (60.9–74.4)	70.8 (54.7–75.2)	74.3 (66.5–80.7)	70.8 (62.1–78.7)	70.8 (60.9–76.8)	0.100
Male sex	36/73 (49%)	19/24 (79%)	8/22 (36%)	11/26 (42%)	74/145 (51%)	0.016
BMI, kg/m^2^	26.3 (24.0–29.1)	24.8 (21.0–27.2)	24.8 (23.0–26.0)	27.5 (24.9–30.3)	26.0 (23.2–28.7)	0.033
ASA ≥ III	45/72 (62%)	12/23 (52%)	14/21 (67%)	12/22 (55%)	83/138 (60%)	0.697
Arterial hypertension	45/72 (62%)	8/24 (33%)	14/21 (67%)	14/26 (54%)	81/143 (57%)	0.064
Diabetes mellitus	18/72 (25%)	8/24 (33%)	5/21 (24%)	3/26 (12%)	34/143 (24%)	0.332
COPD	5/72 (7%)	1/23 (4%)	1/21 (5%)	2/26 (8%)	9/142 (6%)	0.947
Coronary artery disease	10/72 (14%)	4/24 (17%)	2/21 (10%)	3/26 (12%)	19/143 (13%)	0.899
Chronic kidney disease	10/72 (14%)	3/24 (12%)	2/21 (10%)	3/26 (12%)	18/143 (13%)	0.957
Bilirubin, mg/dL	0.5 (0.4–0.8)	2.8 (0.7–4.6)	0.9 (0.5–2.1)	0.5 (0.4–1.0)	0.6 (0.4–1.2)	<0.001
Albumin, g/L	42.4 (39.0–45.5)	37.0 (31.5–41.1)	37.0 (34.4–42.1)	42.5 (37.9–44.5)	41.1 (37.2–44.4)	<0.001
CA 19-9, U/mL	38.0 (11.2–206.0)	178 (55–612)	38.4 (22.4–233.9)	33.7 (10.8–352.8)	48.8 (14.7–329.0)	0.092
Neoadjuvant chemotherapy	15/69 (22%)	1/24 (4%)	2/21 (10%)	3/25 (12%)	21/139 (15%)	0.151
Adjuvant chemotherapy	42/64 (66%)	9/21 (43%)	12/18 (67%)	17/23 (74%)	80/126 (63%)	0.162

**Table 2:** Postoperative morbidity (Clavien–Dindo) by tumor subtype.

Subtype	Patients	Major (≥IIIa)	Grade V
iCCA	73	23/73 (32%, 95% CI 22–43)	5/73 (7%, 95% CI 3–15)
pCCA	24	16/24 (67%, 95% CI 47–82)	8/24 (33%, 95% CI 18–53)
dCCA	22	8/22 (36%, 95% CI 20–57)	3/22 (14%, 95% CI 5–33)
GBC	26	7/26 (27%, 95% CI 14–46)	1/26 (4%, 95% CI 1–19)
**All**	**145**	**54/145 (37%, 95% CI 30–45)**	**17/145 (12%, 95% CI 7–18)**

Note: Grade V counts represent documented events within 90 days. Complete postoperative day-90 vital-status ascertainment was available for 141 of 145 patients; four patients were discharged alive and subsequently lacked reliable follow-up before day 90. The Grade V proportions are therefore documented-event proportions rather than complete 90-day mortality estimates. Bold type indicates the overall cohort.

**Table 3:** Pathological features by subtype among evaluable patients (UICC 8th edition).

Subtype	Patients	T3–4	Node-Positive	Margin Status	G3–4
iCCA	73	9/61 (15%)	22/61 (36%)	R0 30/57 (53%) R1 25/57 (44%) R2 2/57 (4%)	13/43 (30%)
pCCA	24	7/20 (35%)	7/20 (35%)	R0 9/18 (50%) R1 9/18 (50%) R2 0/18 (0%)	7/17 (41%)
dCCA	22	5/17 (29%)	10/16 (62%)	R0 6/15 (40%) R1 9/15 (60%) R2 0/15 (0%)	6/10 (60%)
GBC	26	13/23 (57%)	15/20 (75%)	R0 8/18 (44%) R1 8/18 (44%) R2 2/18 (11%)	5/14 (36%)
**All**	**145**	**34/121 (28%)**	**54/117 (46%)**	**R0 53/108 (49%)** **R1 51/108 (47%)** **R2 4/108 (4%)**	**31/84 (37%)**

Bold type indicates the overall cohort.

**Table 4:** Molecular testing by tumor subtype.

Subtype	*n*	Profiled	NGS Eval	FISH Eval	MSI Eval	≥1 NGS Alt.
iCCA	73	61 (84%)	61	46	56	33/61 (54%)
pCCA	24	15 (62%)	15	13	14	10/15 (67%)
dCCA	22	14 (64%)	14	12	13	6/14 (43%)
GBC	26	13 (50%)	12	10	12	4/12 (33%)
**All**	**145**	**103 (71%)**	**102**	**81**	**95**	**53/102 (52%)**

Bold type indicates the overall cohort.

**Table 5:** Genomic alteration prevalence using assay-specific evaluable denominators. Values are *n*/*N* (%).

Alteration	iCCA	pCCA	dCCA	GBC	Overall
IDH1	13/61 (21.3%)	0/15 (0.0%)	0/14 (0.0%)	0/12 (0.0%)	13/102 (12.7%)
IDH2	3/61 (4.9%)	0/15 (0.0%)	0/14 (0.0%)	0/12 (0.0%)	3/102 (2.9%)
FGFR2 alteration (NGS) *	2/61 (3.3%)	0/15 (0.0%)	0/14 (0.0%)	0/12 (0.0%)	2/102 (2.0%)
FGFR2 rearrangement (FISH)	7/46 (15.2%)	0/13 (0.0%)	0/12 (0.0%)	0/10 (0.0%)	7/81 (8.6%)
KRAS	11/61 (18.0%)	6/15 (40.0%)	4/14 (28.6%)	0/12 (0.0%)	21/102 (20.6%)
NRAS	4/61 (6.6%)	1/15 (6.7%)	0/14 (0.0%)	0/12 (0.0%)	5/102 (4.9%)
BRAF	3/61 (4.9%)	1/15 (6.7%)	0/14 (0.0%)	0/12 (0.0%)	4/102 (3.9%)
ERBB2	1/61 (1.6%)	2/15 (13.3%)	0/14 (0.0%)	1/12 (8.3%)	4/102 (3.9%)
PIK3CA	4/61 (6.6%)	0/15 (0.0%)	2/14 (14.3%)	2/12 (16.7%)	8/102 (7.8%)
TP53 †	0 detected	1 detected	1 detected	1 detected	3 detected
MSI-high	0/56 (0.0%)	2/14 (14.3%)	1/13 (7.7%)	0/12 (0.0%)	3/95 (3.2%)

* The NGS FGFR2 category includes reportable NGS-detected FGFR2 alterations; FISH-detected FGFR2 rearrangements are reported separately. † TP53 was not included in the Oncomine Focus Assay and was therefore not uniformly evaluable across the cohort; counts are shown descriptively and no cross-cohort negative prevalence denominator is presented.

**Table 6:** Median overall survival in the full cohort by anatomical subtype (months; NR = not reached).

Group	Patients	Median OS
iCCA	73	42.2
pCCA	24	17.8
dCCA	22	NR
GBC	26	61.8
Whole cohort	145	40.0

Overall-survival log-rank *p* = 0.250.

## Data Availability

The data presented in this study are available from the corresponding author upon reasonable request due to institutional data protection policies.

## Supplementary Materials

The following supporting information can be downloaded at https://www.mdpi.com/article/10.3390/cancers18213472/s1: Supplementary Table S1. Availability and missingness of major pathological variables; Supplementary Table S2. Sensitivity analysis among confirmed 90-day survivors; Supplementary Table S3. Derivation of the 90-day survivor sensitivity cohort; Supplementary Table S4. Molecular profiling uptake by anatomical subtype and calendar year; Supplementary Table S5. Gene and alteration-type coverage of the NGS assays used during the study period; Supplementary Table S6. FGFR2 FISH-positive iCCA tumors: Paired NGS and FISH findings; Supplementary Table S7. Molecular characteristics of four KRAS co-altered iCCA tumors; Supplementary Table S8. Conventional recurrence-free survival in the full cohort by anatomical subtype; Supplementary Table S9. Patient-level reportable NGS alterations (*n* = 53); Supplementary Figure S1. Overall survival among confirmed 90-day survivors; Supplementary Figure S2. Recurrence-free survival among confirmed 90-day survivors; Supplementary Figure S3. Conventional recurrence-free survival in the full cohort; Supplementary Figure S4. Exploratory R0-versus-R1 sensitivity analysis; Supplementary Figure S5. Representative FGFR2 break-apart FISH images.

## Abbreviations

ASA	American Society of Anesthesiologists
BTC	biliary tract cancer
dCCA	distal cholangiocarcinoma
FISH	fluorescence in situ hybridization
GBC	gallbladder cancer
iCCA	intrahepatic cholangiocarcinoma
MSI	microsatellite instability
NGS	next-generation sequencing
OS	overall survival
pCCA	perihilar cholangiocarcinoma
RFS	recurrence-free survival
UICC	Union for International Cancer Control
