# Supplementary material for: Molecular and Clinical Heterogeneity in Resected Biliary Tract Cancer: A Single-Center Analysis of Treatment Patterns and Oncologic Outcomes
Source: Cancers (Basel). 2026 Oct 27;18(21):3472. doi: 10.3390/cancers18213472 (PMC13761758; doi:10.3390/cancers18213472)
Supplement: Supplementary file 1 [file cancers-18-03472-s001.zip › cancers-4578075-supplementary.pdf]

Supplementary Materials

Molecular and Clinical Heterogeneity in Resected Biliary Tract Cancer

Supplementary Table S1. Availability and missingness of major pathological variables

Variable	Available, n (%)	Missing, n (%)	Total
T category	121 (83.4%)	24 (16.6%)	145
Lymph node status	117 (80.7%)	28 (19.3%)	145
Resection margin status	108 (74.5%)	37 (25.5%)	145
Histological grade	84 (57.9%)	61 (42.1%)	145

No missing values were imputed. Variable-specific analyses therefore use the number of patients with available data as the denominator.

Supplementary Table S2. Sensitivity analysis among confirmed 90-day survivors

Group	Patients	Median OS, months	Median RFS, months
iCCA	66	52.1	13.6
pCCA	15	NR	NR
dCCA	19	NR	19.0
GBC	24	61.8	13.3
Whole survivor cohort	124	53.8	14.0

Overall-survival log-rank p = 0.697; recurrence-free-survival log-rank p = 0.308. This sensitivity cohort included patients with confirmed vital status who were alive at postoperative day 90 (n = 124). Survival time remains measured from the date of index resection.

Supplementary Table S3. Derivation of the 90-day survivor sensitivity cohort

Step	n	Explanation
Index cohort	145	All unique patients undergoing curative-intent index BTC resection.
Documented Grade V events within 90 days	17	5 iCCA, 8 pCCA, 3 dCCA, 1 GBC.
Vital status at day 90 not confirmable	4	Discharged alive; returned to countries of residence abroad and had no reliable follow-up before postoperative day 90.
Eligible 90-day survivors	124	Included in the complementary sensitivity analysis.

Supplementary Table S4. Molecular profiling uptake by anatomical subtype and calendar year

Stratum	Total patients	Profiled, n (%)	Not profiled, n (%)
iCCA	73	61 (84%)	12 (16%)
pCCA	24	15 (62%)	9 (38%)
dCCA	22	14 (64%)	8 (36%)
GBC	26	13 (50%)	13 (50%)
2019	18	11 (61%)	7 (39%)
2020	30	22 (73%)	8 (27%)
2021	19	14 (74%)	5 (26%)
2022	15	12 (80%)	3 (20%)
2023	8	6 (75%)	2 (25%)

2024	33	25 (76%)	8 (24%)
2025	22	13 (59%)	9 (41%)

Profiling uptake differed significantly by anatomical subtype (chi-squared p = 0.006) and ranged from 59% to 80% across calendar years (chi-squared p = 0.740). These differences highlight potential selection bias in the molecularly profiled subgroup, which should not be considered representative of the entire surgical cohort. Additional clinicopathological comparisons between profiled and non-profiled patients were not performed because some historical variables could not be consistently linked at the patient level.

Supplementary Figure S1. Overall survival among confirmed 90-day survivors

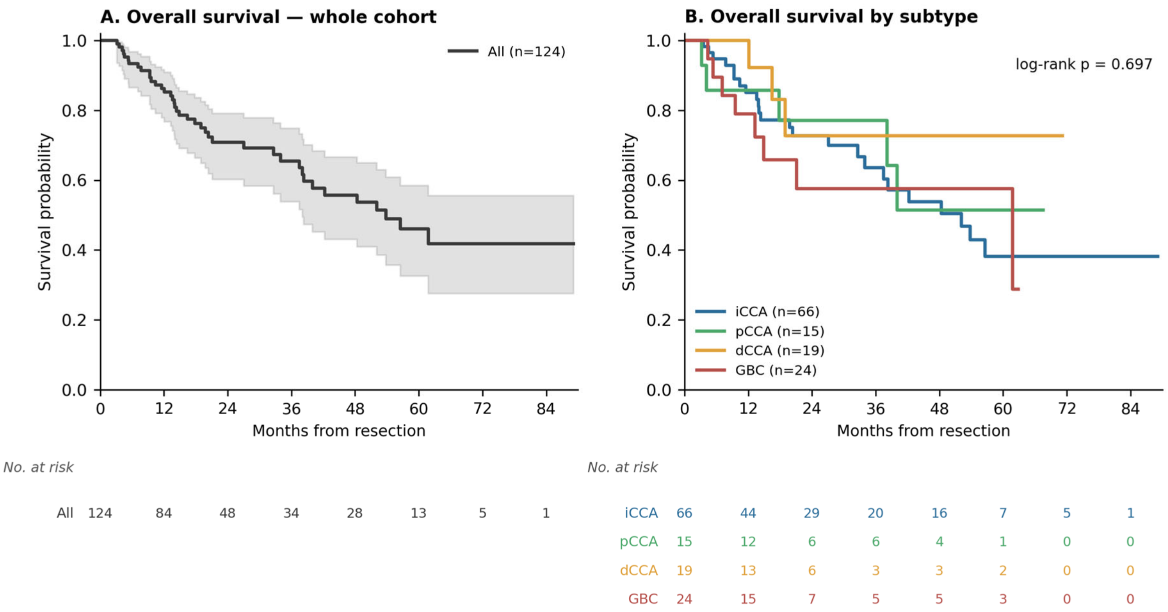

Kaplan–Meier sensitivity analysis restricted to patients with confirmed survival to postoperative day 90 (n = 124); time is measured from the date of index resection.

Supplementary Figure S2. Recurrence-free survival among confirmed 90-day survivors

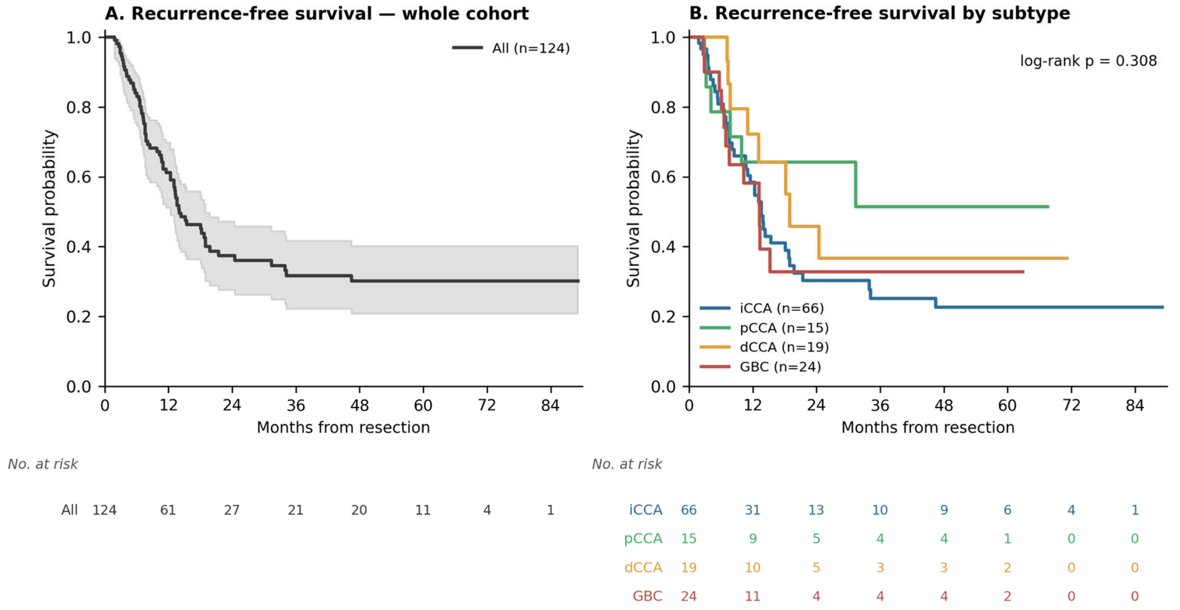

Kaplan–Meier sensitivity analysis restricted to patients with confirmed survival to postoperative day 90 (n = 124); time is measured from the date of index resection.

Supplementary Table S5. Gene and alteration-type coverage of the NGS assays used during the study period

Assay	Alteration type	Genes
Oncomine Focus Assay	Hotspot / SNV-indel	AKT1, ALK, AR, BRAF, CDK4, CTNNB1, DDR2, EGFR, ERBB2, ERBB3, ERBB4, ESR1, FGFR2, FGFR3, GNA11, GNAQ, HRAS, IDH1, IDH2, JAK1, JAK2, JAK3, KIT, KRAS, MAP2K1, MAP2K2, MET, MTOR, NRAS, PDGFRA, PIK3CA, RAF1, RET, ROS1, SMO
Oncomine Focus Assay	Copy-number alteration	ALK, AR, BRAF, CCND1, CDK4, CDK6, EGFR, ERBB2, FGFR1, FGFR2, FGFR3, FGFR4, KIT, KRAS, MET, MYC, MYCN, PDGFRA, PIK3CA
Oncomine Focus Assay	Fusion / rearrangement	ABL1, AKT3, ALK, AXL, BRAF, EGFR, ERBB2, ERG, ETV1, ETV4, ETV5, FGFR1, FGFR2, FGFR3, MET, NTRK1, NTRK2, NTRK3, PDGFRA, PPARG, RAF1, RET, ROS1
Oncomine Precision / Genexus	Hotspot / SNV-indel	AKT1, AKT2, AKT3, ALK, AR, ARAF, BRAF, CDK4, CDKN2A, CHEK2, CTNNB1, EGFR, ERBB2, ERBB3, ERBB4, ESR1, FGFR1, FGFR2, FGFR3, FGFR4, FLT3, GNA11, GNAQ, GNAS, HRAS, IDH1, IDH2, KIT, KRAS, MAP2K1, MAP2K2, MET, MTOR, NRAS, NTRK1, NTRK2, NTRK3, PDGFRA, PIK3CA, PTEN, RAF1, RET, ROS1, SMO, TP53
Oncomine Precision / Genexus	Copy-number alteration	ALK, AR, CD274, CDKN2A, EGFR, ERBB2, ERBB3, FGFR1, FGFR2, FGFR3, KRAS, MET, PIK3CA, PTEN
Oncomine Precision / Genexus	Inter-gene fusion	ALK, BRAF, ESR1, FGFR1, FGFR2, FGFR3, MET, NRG1, NTRK1, NTRK2, NTRK3, NUTM1, RET, ROS1, RSPO2, RSPO3
Oncomine Precision / Genexus	Intra-gene fusion	AR, EGFR, MET

Interpretive note: TP53 is included in Precision/Genexus but not in OFA. ARID1A, BAP1, and PBRM1 are not included in either assay used in this cohort and therefore cannot be interpreted as tested-negative genes.

Supplementary Table S6. FGFR2 FISH-positive iCCA tumors: Paired NGS and FISH findings

Case ID	Year	NGS assay	FGFR2 NGS	FGFR2 FISH	Other reportable NGS finding
F01	2020	Oncomine Focus	Negative	Positive	None reported
F02	2021	Oncomine Focus	Negative	Positive	None reported
F03	2021	Oncomine Focus	Negative	Positive	None reported
F04	2021	Oncomine Focus	Negative	Positive	KRAS p.Gly12Val
F05	2023	Oncomine Focus	Negative	Positive	None reported
F06	2024	Oncomine Focus	Negative	Positive	None reported
F07	2024	Oncomine Focus	FGFR2(17)-CASP7(2) fusion	Positive	KRAS p.Gln61His

Interpretation: Six of seven FISH-positive iCCA tumors had no reportable FGFR2 event by targeted NGS, whereas one had a concordant FGFR2–CASP7 fusion call. Limited targeted-NGS fusion coverage or RNA quality may contribute to discordance, but atypical or false-positive FISH interpretations are also possible. FGFR2 break-apart FISH identifies rearrangement-associated signals without determining the fusion partner or establishing an expressed functional fusion transcript. Without further orthogonal testing, the direction of discordance remains unresolved.

Supplementary Table S7. Molecular characteristics of four KRAS co-altered iCCA tumors

Case ID	Year	NGS assay	KRAS alteration	Co-alteration	FGFR2 FISH
K01	2020	Oncomine Focus	p.Val14Ile	IDH1 p.Arg132Gly	Not performed
K02	2021	Oncomine Focus	p.Gly12Asp	IDH1 p.Arg132Cys	Not performed
K03	2024	Oncomine Focus	p.Gln61His	FGFR2(17)-CASP7(2) fusion	Positive
K04	2025	Oncomine Precision / Genexus	p.Gln61Arg	IDH1 p.Arg132Cys	Negative

Interpretation: Four iCCA tumors exhibited co-detected KRAS alterations with IDH1 or FGFR2 findings across both assay periods. The KRAS/FGFR2 case was also FGFR2 FISH-positive. These uncommon findings are presented descriptively without inferring a shared clonal origin or distinct biological mechanism.

Supplementary Table S8. Conventional recurrence-free survival in the full cohort by anatomical subtype

Group	Patients	Median RFS, months
iCCA	73	12.4
pCCA	24	4.1
dCCA	22	18.2
GBC	26	13.2

Overall subtype comparison: log-rank $p = 0.806$. RFS was defined as time from the index resection to first documented recurrence or death from any cause, whichever occurred first.

Supplementary Figure S3. Conventional recurrence-free survival in the full cohort

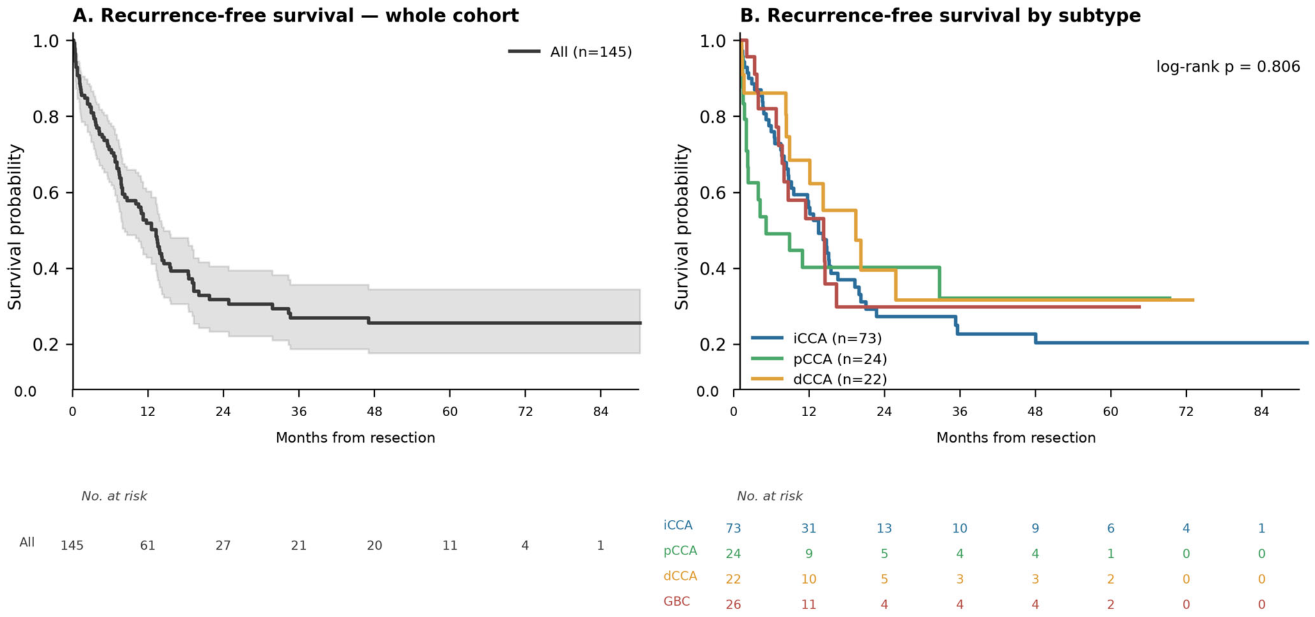

Kaplan–Meier analysis from the date of index resection in the full 145-patient cohort. Recurrence or death from any cause was counted as an RFS event.

Supplementary Figure S4. Exploratory R0-versus-R1 sensitivity analysis

A

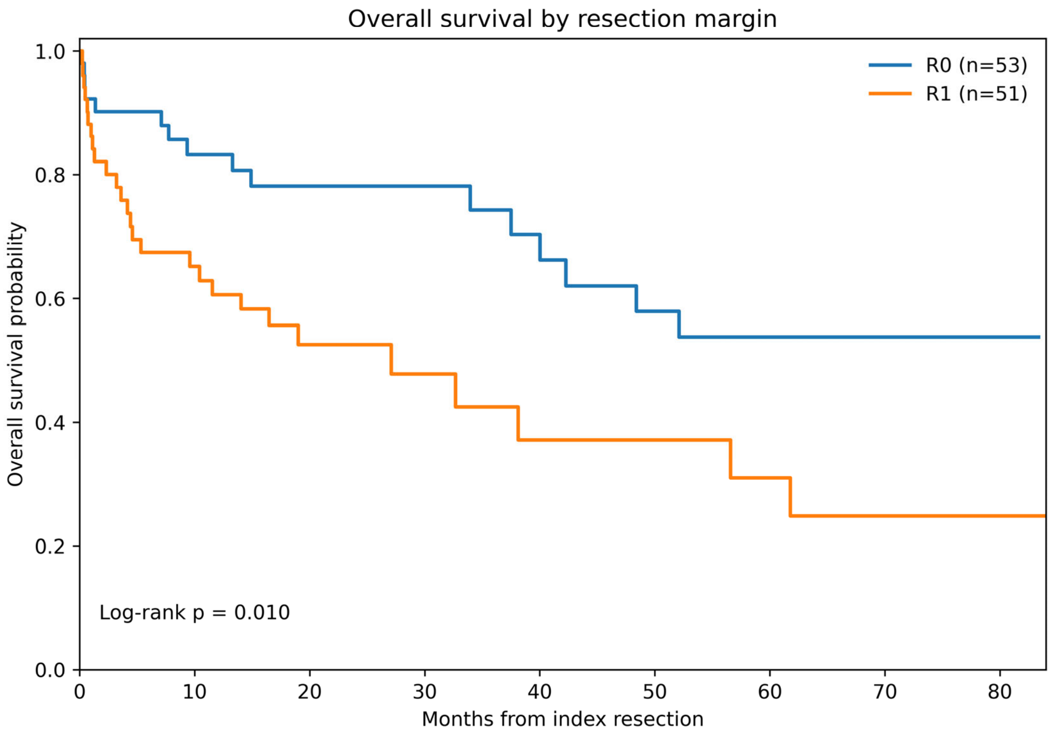

B

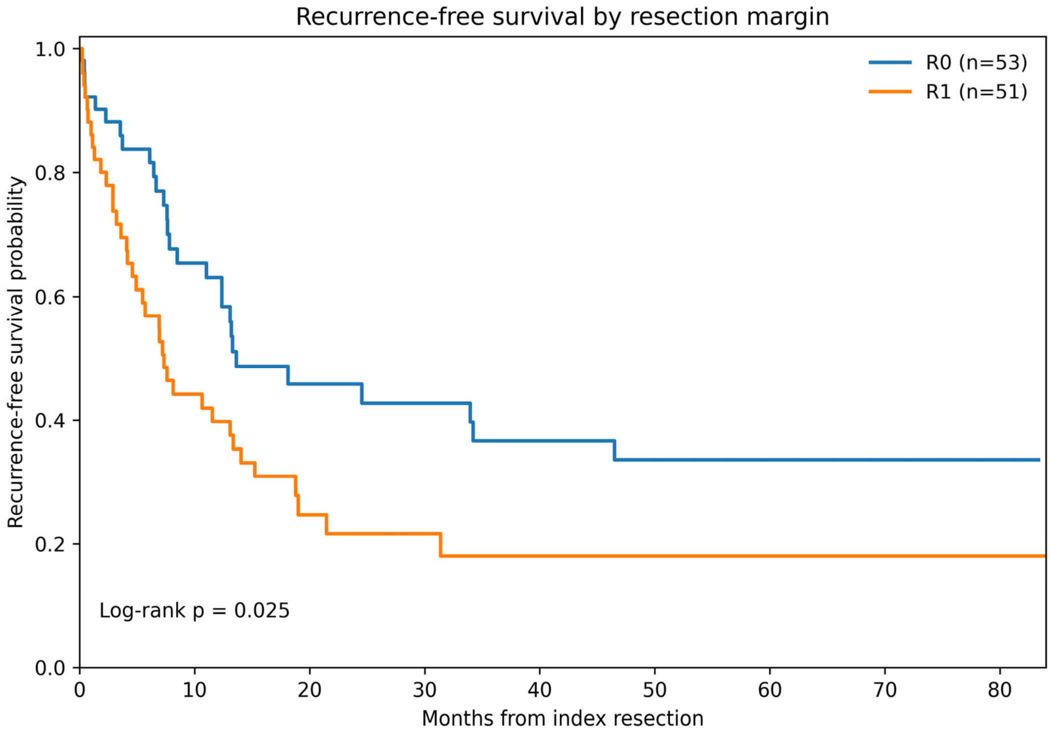

Kaplan–Meier sensitivity analyses among patients with evaluable margin status, excluding the four R2 resections. (A) Overall survival by margin status (R0, n = 53; R1, n = 51; log-rank p = 0.010). (B) Conventional recurrence-free survival by margin status (R0, n = 53; R1, n = 51; log-rank p = 0.025).

Supplementary Table S9. Patient-level reportable NGS alterations (n = 53)

Study ID	BTC subtype	MP accession year	NGS panel	Pathology-reported alteration(s) [event class]
S003 (K02)	iCCA	2021	OFA	IDH1 p.Arg132Cys (missense variant); KRAS p.Gly12Asp (missense variant)
S005	iCCA	2020	OFA	BRAF p.Asn581Ile (missense variant)
S007 (K01)	iCCA	2020	OFA	IDH1 p.Arg132Gly (missense variant); KRAS p.Val14Ile (missense variant)
S009	iCCA	2021	OFA	NRAS p.Gln61Arg (missense variant)
S012	pCCA	2019	OFA	KRAS p.Gly12Ala (missense variant)
S024	pCCA	2020	OFA	NRAS p.Gln61Lys (missense variant)
S026	iCCA	2020	OFA	PIK3CA p.Glu542Gly (missense variant); FGFR2 p.Pro263_Ala266del (in-frame deletion (protein-level))
S027	iCCA	2020	OFA	KRAS p.Gly12Val (missense variant); PIK3CA p.Glu545Lys (missense variant)
S028	iCCA	2021	OFA	IDH1 p.Arg132Cys (missense variant)
S030	iCCA	2020	OFA	NRAS p.Gln61Arg (missense variant)
S031	iCCA	2020	OFA	KRAS p.Gly12Arg (missense variant)
S036	iCCA	2020	OFA	IDH1 p.Arg132Cys (missense variant)
S037	pCCA	2020	OFA	BRAF p.Lys641Glu (missense variant); HRAS p.Gly12Asp (missense variant)
S048	iCCA	2020	OFA	IDH1 p.Arg132Cys (missense variant)
S050	pCCA	2021	OFA	KRAS p.Gly12Asp (missense variant)
S052	iCCA	2021	OFA	IDH1 p.Arg132Leu (missense variant)
S053	dCCA	2021	OFA	KRAS p.Gln61His (missense variant); PIK3CA p.His1047Arg (missense variant)
S055	iCCA	2020	OFA	ERBB2 Amplification (amplification)
S057	iCCA	2021	OFA	BRAF p.Lys601Glu (missense variant)
S058	iCCA	2021	OFA	NRAS p.Gln61Arg (missense variant)
S061	GBC	2022	OFA	EGFR Amplification (amplification)
S065	iCCA	2021	OFA	IDH1 p.Arg132Cys (missense variant)
S067	pCCA	2021	OFA	KRAS p.Gln61His (missense variant); GNA11 p.Arg183Cys (missense variant)
S069	pCCA	2022	OFA	KRAS p.Gly12Val (missense variant)
S070	iCCA	2022	OFA	IDH1 p.Arg132His (missense variant); BRAF p.Lys601Glu (missense variant)
S074	pCCA	2022	OFA	ERBB2 Amplification (amplification)
S075	iCCA	2021	OFA	KRAS p.Gly12Val (missense variant)
S076	iCCA	2022	OFA	IDH1 p.Arg132Cys (missense variant)
S077	GBC	2023	OFA	PIK3CA residue 545→Lys (missense; reference residue unknown)
S079	iCCA	2022	OFA	KRAS p.Gly12Cys (missense variant)
S080	iCCA	2023	OFA	KRAS p.Gly12Asp (missense variant)
S083	pCCA	2023	OFA	KRAS p.Gly12Cys (missense variant)
S086	iCCA	2021	OFA	KRAS p.Gly12Asp (missense variant)
S090	pCCA	2024	OFA	KRAS p.Gly12Cys (missense variant)
S091	dCCA	2024	OFA	KRAS p.Gly12Val (missense variant)
S092	dCCA	2024	OFA	KRAS p.Gly12Val (missense variant); CDK6 Amplification (amplification)
S094	iCCA	2024	OFA	IDH1 p.Arg132Cys (missense variant); PIK3CA p.Glu542Lys (missense variant)

Study ID	BTC subtype	MP accession year	NGS panel	Pathology-reported alteration(s) [event class]
S096	iCCA	2024	OFA	IDH1 p.Arg132Cys (missense variant)
S098	dCCA	2024	OFA	KRAS p.Gly12Cys (missense variant)
S102	iCCA	2024	OFA	IDH2 p.Arg172Gly (missense variant)
S104	iCCA	2024	OFA	IDH2 p.Arg172Gly (missense variant)
S105	dCCA	2024	OFA	PIK3CA p.Glu542Lys (missense variant)
S107	GBC	2024	OFA	PIK3CA p.Glu545Lys (missense variant); ERBB2 Amplification (amplification)
S108	iCCA	2024	OFA	NRAS p.Gln61Arg (missense variant)
S112	iCCA	2024	OFA	IDH2 p.Arg172Gly (missense variant)
S118 (K03 / F07)	iCCA	2024	OFA	KRAS p.Gln61His (missense variant); FGFR2(17) - CASP7(2) (fusion)
S121	iCCA	2024	Precision / Genexus	IDH1 p.Arg132Cys (missense variant)
S134	pCCA	2025	Precision / Genexus	TP53 p.Arg175His (missense variant); ERBB2 amplification (amplification)
S135	iCCA	2025	Precision / Genexus	KRAS p.Gln61Lys (missense variant)
S138	iCCA	2025	Precision / Genexus	PIK3CA p.Met1043Val (missense variant)
S141	dCCA	2025	Precision / Genexus	CTNNB1 p.Lys335Ile (missense variant); TP53 p.Ile255Phe (missense variant)
S142 (K04)	iCCA	2025	Precision / Genexus	IDH1 p.Arg132Cys (missense variant); KRAS p.Gln61Arg (missense variant)
S143	GBC	2025	Precision / Genexus	TP53 p.Arg175His (missense variant)

OFA, Oncomine Focus Assay; Precision/Genexus, Oncomine Precision assay on Genexus; BTC, biliary tract cancer. Only alteration-positive patients are displayed.

Supplementary Figure S5. Representative FGFR2 break-apart FISH images

A

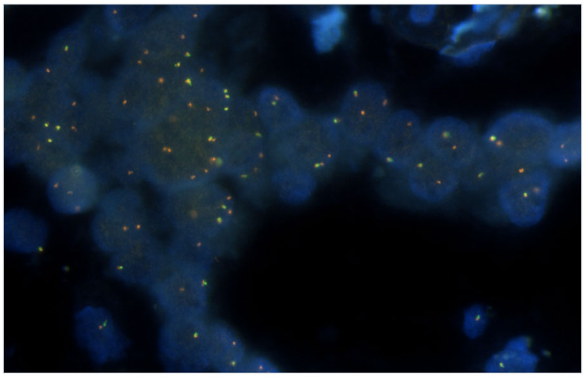

B

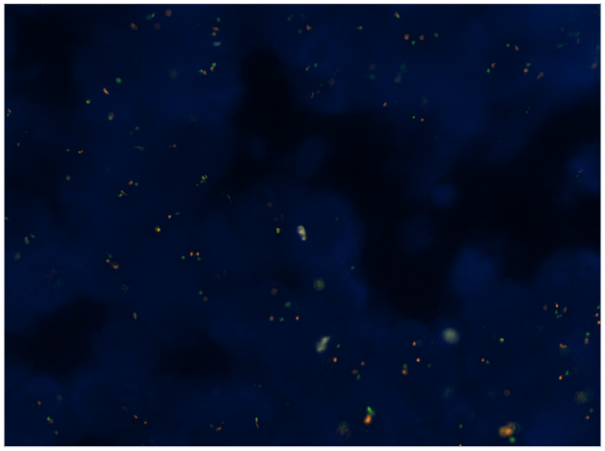

A, FISH-positive and targeted NGS-positive iCCA with FGFR2(17)–CASP7(2) fusion; B, representative field interpreted as FISH-positive but RNA-targeted-NGS-negative. Photographs illustrate representative observations.
